# Effects of long-term childhood exercise and detraining on lipid accumulation in metabolic-related organs

**DOI:** 10.1371/journal.pone.0270330

**Published:** 2022-06-24

**Authors:** Son Tien Nguyen, Naoto Fujita, Takaya Oshima, Misuzu Nishihira, Haruya Ohno, Masayasu Yoneda, Susumu Urakawa

**Affiliations:** 1 Department of Musculoskeletal Functional Research and Regeneration, Graduate School of Biomedicine and Health Sciences, Hiroshima University, Kasumi, Minami-ku, Hiroshima, Japan; 2 Department of Molecular and Internal Medicine, Graduate School of Biomedical and Health Sciences, Hiroshima University, Kasumi, Minami-ku, Hiroshima, Japan; 3 Department of Preventive Medicine for Diabetes and Lifestyle-Related Diseases, Graduate School of Biomedical and Health Sciences, Hiroshima University, Kasumi, Minami-ku, Hiroshima, Japan; Medical University of Vienna, AUSTRIA

## Abstract

The preventive effects of regular exercise on obesity-related health problems are carried over to the non-exercise detraining period, even when physical activity decreases with aging. However, it remains unknown whether regular childhood exercises can be carried over to adulthood. Therefore, this study aimed to investigate the effects of long-term childhood exercise and detraining on lipid accumulation in organs to prevent obesity in adulthood. Four-week-old male Otsuka Long-Evans Tokushima Fatty (OLETF) rats were used as obese animals. OLETF rats were allocated into sedentary and exercise groups: exercise from 4- to 12-week-old and detraining from 12- to 20-week-old. At 12-week-old immediately after the exercise period, regular exercise completely inhibited hyperphagia, obesity, enlarged pancreatic islets, lipid accumulation and lobular inflammation in the liver, hypertrophied adipocytes in the white adipose tissue (WAT), and brown adipose tissue (BAT) whitening in OLETF rats. Additionally, exercise attenuated the decrease in the ratio of muscle wet weight to body weight associated with obesity. Decreased food consumption was maintained during the detraining period, which inhibited obesity and diabetes at 20-week-old after the detraining period. Histologically, childhood exercise inhibited the enlargement of pancreatic islets after the detraining period. In addition, inhibition of lipid accumulation was completely maintained in the WAT and BAT after the detraining period. However, the effectiveness was only partially successful in lipid accumulation and inflammation in the liver. The ratio of muscle wet weight to body weight was maintained after detraining. In conclusion, early long-term regular exercise effectively prevents obesity and diabetes in childhood, and its effectiveness can be tracked later in life. The present study suggests the importance of exercise during childhood and adolescence to inhibit hyperphagia-induced lipid accumulation in metabolic-related organs in adulthood despite exercise cessation.

## Introduction

Childhood obesity is a non-communicable disease that has drastically increased in prevalence worldwide, with an increase from 30 million children under aged 5 years affected in 2000 to nearly 39 million in 2020 [1]. Furthermore, childhood obesity is usually carried over to adulthood, with a 1.3 fold increase in the risk of developing adulthood obesity compared with non-obese children [2]. In addition, children with obesity have a higher risk of developing metabolic disorders and disabilities, such as diabetes, in later life [3]. Therefore, the treatment and prevention of childhood obesity are of particular importance.

Clinically, treatment for childhood obesity aims to alleviate or reverse lipid accumulation in the whole body, with lifestyle modification being the cornerstone [4]. Exercise is effective in preventing and treating childhood obesity [5]. However, children who are overweight and obese tend to have trouble adhering to a continual exercise program, with a high dropout rate of approximately 50% [6]. In addition, strict adherence to exercise regimens is difficult for children due to injuries, determination, desire, and design of exercise programs [7], or recently, the coronavirus disease 2019 (COVID-19) lock-down. Therefore, discontinuation of exercise could induce a detraining effect, which may negatively impact children’s metabolic health.

In adults with obesity, detraining has been shown to partly or totally attenuate the metabolic benefits of exercise, with a significant increase in lipid accumulation, insulin resistance, and insulin levels [8, 9]. A study by Weinsier *et al*. showed that in postmenopausal obese women, although exercise could reduce their body weight to the level matched to lean counterparts, detraining increased their body weight significantly compared to lean counterparts [8]. Fatouros *et al*. also found that 6-month detraining following 6-month exercise in obese men reversed body mass index, leptin, adiponectin, glucose, and insulin levels to the baseline measurements [9]. In contrast, in children with obesity, long-term exercise intervention could prolong its effectiveness in preventing obesity and obesity-related metabolic complications. A study by Garcia-Hermoso *et al*. involving children with obesity aged 8 to 11 years outlined that 3-year exercise training could sustain low %fat mass and body mass index after 6-months of detraining [10]. Another study by Reinehr *et al*. involving children with obesity aged 6 to 14 years outlined that 1-year exercise training could sustain low body mass index, insulin levels, and insulin resistance index at 1-year follow-up [11]. However, information regarding lipid accumulation in children with obesity undergoing detraining is limited. Lipid accumulation during the detraining period occurs in the main lipid supply organs, such as the intra-abdominal white adipose tissue (WAT), brown adipose tissue (BAT), and liver. Moreover, lipid accumulation in organs increases insulin resistance, which is similar to a vicious cycle. Therefore, the question arises whether there are unique changes in lipid accumulation in organs under the effects of exercise in childhood. However, studies on lipid accumulation during childhood detraining have mostly focused on individual organs. Hence, there is a need to investigate lipid accumulation in the organs.

Compared to human studies, animal studies have directly examined lipid accumulation in many organs. The Otsuka Long-Evans Tokushima Fatty (OLETF) rat is an obesity model characterized by hyperphagia due to the loss of cholecystokinin receptor-1 in the dorsomedial hypothalamus, leading to decreased satiety [12]. The natural progression of obesity and obesity-related metabolic complications in OLETF rats reflects human childhood obesity, leading to metabolic health problems in adulthood. OLETF rats exhibit an early development of mild obesity after weaning, with overeating and sedentariness, which could be prevented by lifestyle modification with diet and exercise. Obesity in OLETF rats gradually leads to glucose intolerance, and insulin resistance at around 12-week-old and then diabetes and chronic obesity complications occur no earlier than 20-week-old (or adulthood), similar to those of human obesity [13]. Therefore, OLETF rats are a suitable model for investigating the influence of exercise on childhood obesity and obesity-related complications later in life. Given the unclear effects of long-term childhood exercise and detraining on lipid accumulation, we conducted a study on young OLETF rats subjected to long-term wheel running. The purpose of the present study was to investigate the influence of long-term childhood exercise and detraining on the progression to obesity, focusing on lipid accumulation in the peripheral organs as a whole and whether the effectiveness of childhood exercise was carried over to adulthood after a period of detraining. These results further confirmed the merits of long-term childhood exercise in preventing obesity and metabolic disorders.

## Materials and methods

### Experimental design

This study was approved by the Institutional Animal Care and Use Committee of Hiroshima University (A19-163) and conducted under the Hiroshima University Regulations for Animal Experimentation. All experiments were performed in accordance with the National Institute of Health Guidelines for the Care and Use of Laboratory Animals. The health of the rats was monitored twice a day. If the rats expressed the specific signs such as severe weight loss, tachypnea, and lethargy, we judged the signs as terminal. The rats that showed the signs (*i*.*e*., rats nearing death) were humanely euthanized by an overdose of sodium pentobarbital to minimize the distress.

Four-week-old male OLETF rats and age-matched male Long-Evans Tokushima Otsuka (LETO) rats were used as the obese and non-obese control groups, respectively. OLETF rats were randomly assigned to the sedentary (OLETF Sed, *n* = 12) and exercise (OLETF Ex, *n* = 12) groups. All rats were housed in three and provided standard chow and water *ad libitum*. The housing conditions were maintained as follows: ambient temperature of 22 ± 2°C and under a 12–12 light-dark cycle, with the light on from 08:00 and off from 20:00.

### Exercise protocol

OLETF rats in the OLETF Ex group ran freely on a running wheel for 12 h/day from 20:00 to 08:00 and returned to their home cages from 08:00 to 20:00. The rats exercise for 8 weeks from 4- to 12-week-old and non-exercise for 8 weeks from 12- to 20-week-old. The rats freely accessed food and water during running time, and ambient temperature and humidity conditions were maintained the same as in their home cages. Running duration, running distance, and average and maximum running velocities were automatically recorded with the magnetic sensor attached to the running cage and stored in a corresponding digital meter during the exercise period (S1 Fig).

### Oral glucose tolerance test

An oral glucose tolerance test (OGTT) was performed 1 week prior to sacrificing at 20-week-old. The rats were fasted for 12 h prior to testing. For each rat, a dose of 2 g glucose/kg body weight was inserted via an esophageal tube. Blood glucose from the lateral caudal vein was measured at four time points during the test using an ACCU-CHECK ST meter (Roche, Tokyo, Japan), including the fasting point, at 30, 60, and 120 min after glucose administration. Blood was aspirated at the fasting point into heparinized microhematocrit tubes and centrifuged at 3000 rpm for 10 min, and the plasma was stored at –80° C until further analyses.

### Measurements of plasma triacylglycerol, free fatty acid, and insulin

Triacylglycerol (TAG), free fatty acid (FFA), and insulin levels during fasting were quantified from the OGTT plasma samples. TAG and FFA levels were measured using spectrophotometric assay kits (290–63701 and 294–63601, respectively; Wako, Osaka, Japan). Insulin levels were measured using an enzyme-linked immunosorbent assay (ELISA) kit (M1101; Morigana, Yokohama, Japan).

### Tissue sampling

All rats in the OLETF Ex group fasted for 5 h before sampling and after the last exercise and were euthanized by an overdose of sodium pentobarbital. The pancreas, gastrocnemius muscle, liver, epididymal white adipose tissue (eWAT), and interscapular BAT were harvested and frozen in nitrogen or fixed in 4% paraformaldehyde in 0.1 M phosphate buffer.

### Histological analysis

The pancreatic head embedded in paraffin was cut into 5 μm and stained with hematoxylin and eosin (HE). Histological images were used to measure the cross-sectional area of the pancreatic islets, and the diameter was calculated from these values. Images of 20–30 pancreatic islets for each rat were counted for measurement.

Serial transverse sections of the muscle belly of the gastrocnemius lateralis were stained for adenosine triphosphatase (ATPase, pH 4.1) and succinate dehydrogenase (SDH) activities to categorize the muscle fibers as type I, IIA, or IIB. The muscle fibers stained darkly with ATPase and SDH were categorized as type I, lightly with ATPase, darkly with SDH were categorized as type IIA, and lightly with both ATPase and SDH were categorized as type IIB. Two random images with >100 muscle fibers were chosen in each superficial and deep layer to measure the cross-sectional area of the muscle fibers.

Paraffin sections of the left lateral lobe of the liver were stained with HE, and the images were used to measure the percentage of liver steatosis and degree of lobular inflammation. The area in which lipid droplets occurred inside the hepatocytes with microvesicular or macrovesicular structures was defined as the liver steatosis area, and four to five images, including portal triads, were used to measure the percentage of liver steatosis. A × 200 image was divided into 100 μm^2^ squares, and the percentage of liver steatosis was calculated as all squares that contained liver steatosis areas per total squares in the section. Lobular inflammation was calculated as the total number of inflammatory foci showing focal mononuclear cells around the vein or artery per section. In addition to histological analysis, the hepatic TAG content was measured using spectrophotometric assay kits (290–63701, Wako). Total lipids from liver samples were extracted according to Folch’s method [14].

Paraffin sections of the eWAT and BAT were stained with HE. Regarding the eWAT histology, images stained with HE were used to measure the adipocytes’ diameter. Three to five random images with approximately 300 adipocytes were selected to measure the adipocytes’ area, and the diameter was calculated from these values. Adipocytes surrounded by mononuclear cells were defined as crown-like structures (CLS), and the number of CLS was calculated as the total number of CLS per section. Histological parameters such as the cross-sectional area, diameter, and the number in the pancreas, skeletal muscle, liver, and eWAT were quantified using ImageJ software (NIH, Bethesda, MD, USA).

### Statistics

The data were expressed as the mean ± standard error (SEM). Two-way analysis of variance (ANOVA) with Bonferroni post hoc test or one-way ANOVA with Tukey’s post hoc test was used to determine significant differences among the groups. Statistical significance was set at *P* <0.05. The data were processed using SPSS software (IBM SPSS Statistics version 19.0, IBM Japan, Tokyo, Japan).

## Results

### Changes in body weight and food consumption

**Fig 1:**
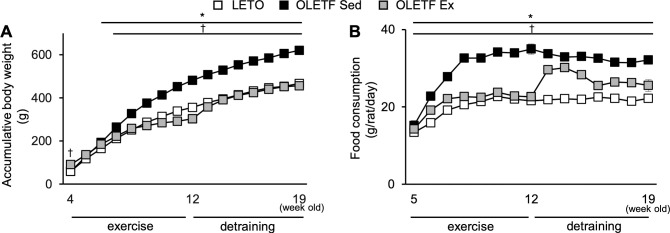
Changes in body weight. Body weight (**A**) and daily food consumption (**B**). Data are illustrated as mean ± SEM. * and † show significantly different between the OLETF Sed group vs. LETO rats and the OLETF Sed vs. OLETF Ex groups, respectively, *P* < 0.05, two-way ANOVA. *n* = 5–6 per group.

From 4- to 5-week-old, the body weight was almost the same between the OLETF Sed group and LETO rats (Fig 1A). Body weight increased weekly, with a steeper growth curve in the OLETF Sed group than in LETO rats. After 6-week-old, the value was significantly higher in the OLETF Sed group than in LETO rats. Food consumption was significantly higher in the OLETF Sed group than in LETO rats throughout the experimental period (Fig 1B).

During the exercise period from 4- to 12-week-old, food consumption was significantly lower in the OLETF Ex group than in the OLETF Sed group. As a result, body weight increased in the OLETF Ex group during the exercise period but significantly lower in the OLETF Ex group than in the OLETF Sed group after 7-week-old.

Food consumption in the OLETF Ex group at 13-week-old (*i*.*e*., a week after the last exercise) was significantly increased compared with that at 12-week-old (*i*.*e*., immediately after the last exercise). However, food consumption in the OLETF Ex group was still significantly lower than that in the OLETF Sed group at all the subsequent time points. In addition, at 13-week-old, the body weight in the OLETF Ex group was significantly higher than that in the 12-week-old but remained lower than that in the OLETF Sed group. The body weight gradually increased in the OLETF Ex group after 14-week-old. However, at the end of the experimental period, the body weight in the OLETF Ex group was still significantly lower than that in the OLETF Sed group.

### Biomedical markers changes

**Fig 2:**
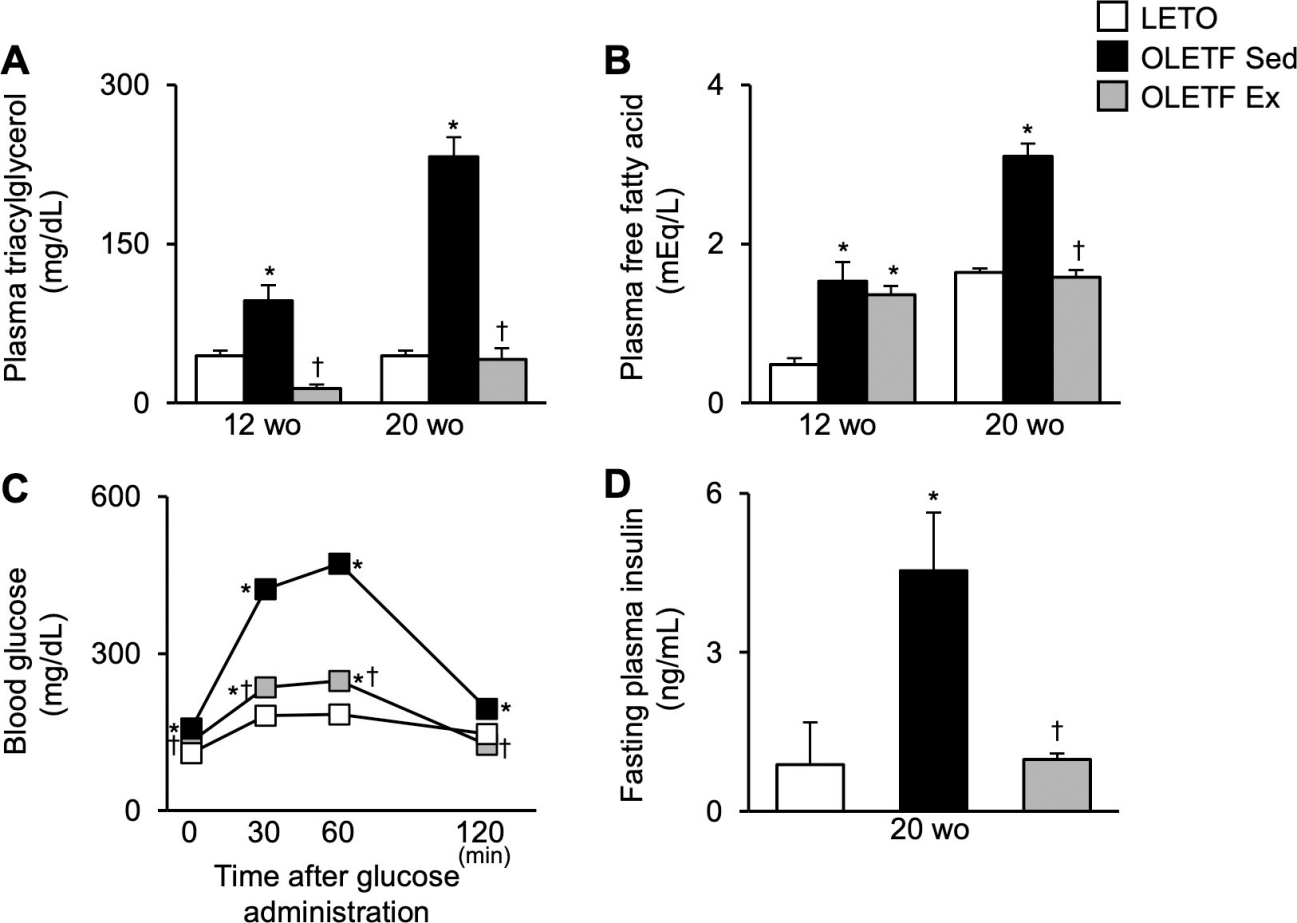
Changes in blood biomarkers. Fasting plasma triacylglycerol (**A**) and free fatty acid (**B**), glucose homeostasis during the oral glucose tolerance test at 20-week-old (**C**), and fasting plasma insulin concentrations at 20-week-old (**D**). wo, week-old. Data are illustrated as mean ± SEM. * and † show significantly different from age-matched LETO rats and age-matched the OLETF Sed group, respectively, *P* < 0.05, one way ANOVA. *n* = 5–6 per group.

Plasma TAG (Fig 2A) and FFA (Fig 2B) levels were significantly higher in the OLETF Sed group than in LETO rats at 12- and 20-week-old. At 12-week-old (*i*.*e*., immediately after the exercise period from 4- to 12-week-old), plasma TAG levels were significantly lower in the OLETF Ex group than in the OLETF Sed group, whereas plasma FFA levels were almost the same between the OLETF Ex and OLETF Sed groups. However, at 20-week-old (*i*.*e*., after the detraining period from 12- to 20-week-old), the values were significantly lower in the OLETF Ex group than in the OLETF Sed group.

Regarding glucose homeostasis at 20-week-old, hyperglycemia was observed in the OLETF Sed group, with significantly higher blood glucose levels at fasting and after glucose administration than in LETO rats (Fig 2C). Although blood glucose levels at fasting and 120 min after glucose administration were significantly higher in the OLETF Sed group than in LETO rats, the values in the OLETF Ex group were significantly lower than those in the OLETF Sed group, almost the same in LETO rats. The values in the OLETF Ex group at 30 and 60 min after glucose administration were also significantly lower than those in the OLETF Sed group but not in LETO rats. Additionally, fasting plasma insulin levels at 20-week-old were significantly higher in the OLETF Sed group than in LETO rats (Fig 2D). These values were significantly lower in the OLETF Ex group than in the OLETF Sed group.

### Histological changes in the pancreas

**Fig 3:**
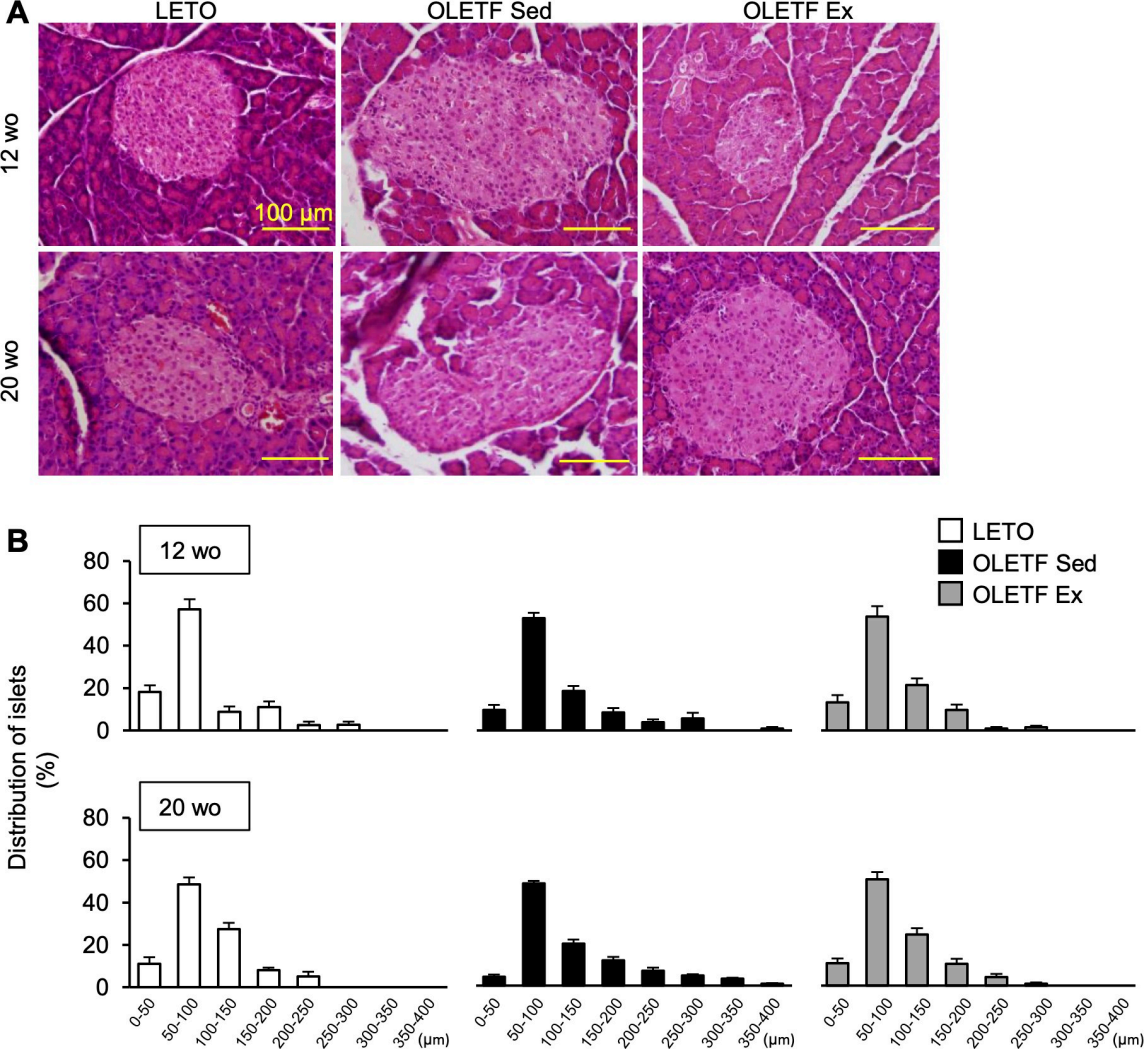
Histological changes in the pancreas. Representative pancreas images stained with hematoxylin and eosin (**A**) and distribution of islets diameter (**B**). wo, week-old. Data are illustrated as mean ± SEM.

At 12-week-old, the islets were oval, and there was no lipid accumulation in the islets of all the groups (Fig 3A). However, large islets with diameters from 350 to 400 μm were observed only in the OLETF Sed group but not in the OLETF Ex group (Fig 3B).

At 20-week-old, distorted large islets with a multilobular shape were observed in the OLETF Sed group, whereas the shape of the large islets was still oval in the OLETF Ex group. In addition, no lipid accumulation was observed in the islets of all groups (Fig 3A). Huge islets with diameters ranging from 350 to 400 μm were still observed only in the OLETF Sed group, and no islets in the OLETF Ex group exceeded 300 μm in diameter. (Fig 3B).

### Histological changes in the skeletal muscle

**Fig 4:**
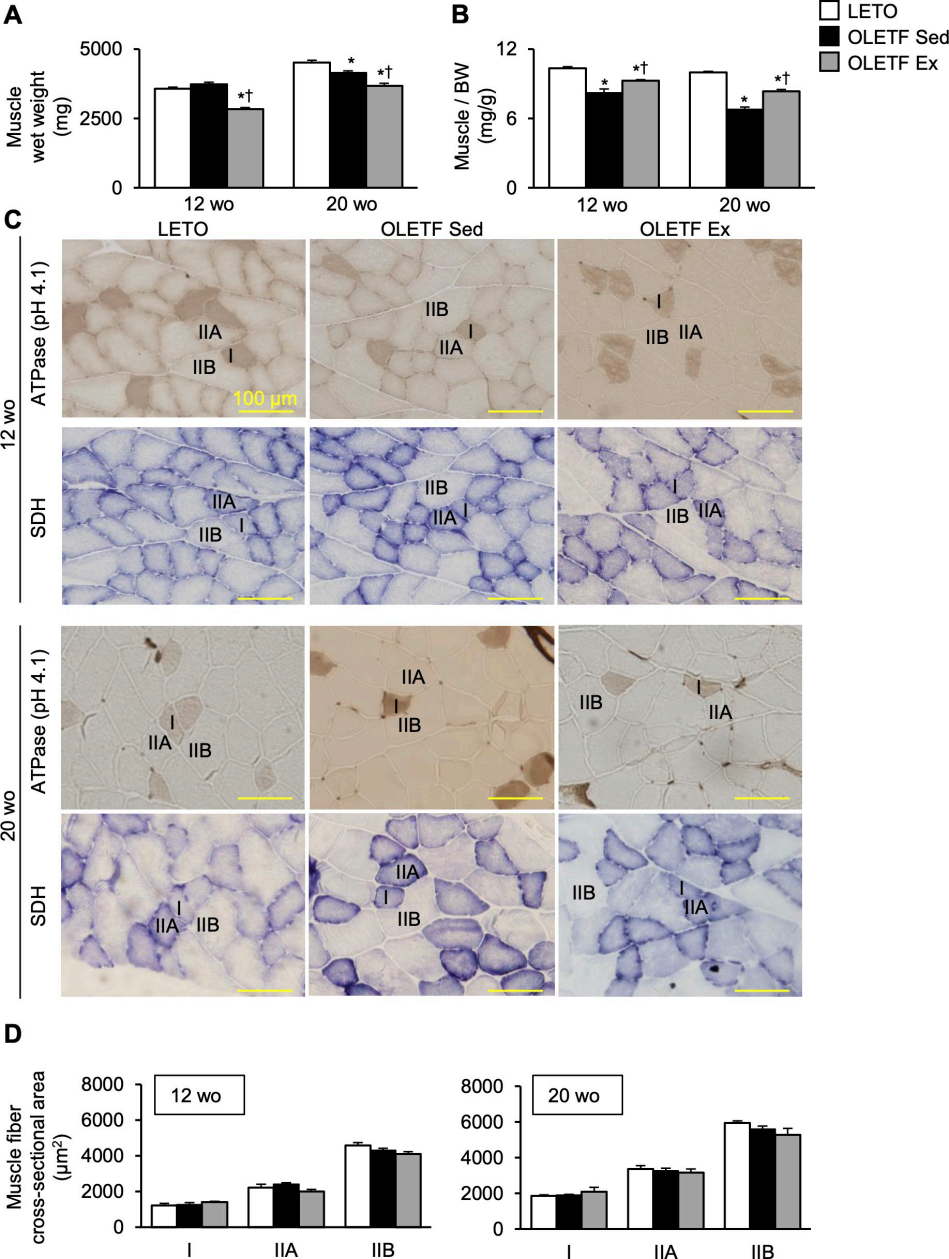
Histological changes in the skeletal muscle. Gastrocnemius muscle wet weight (**A**), the ratio of muscle wet weight to body weight (**B**), representative gastrocnemius lateralis images (**C**), and cross-sectional area of each muscle fiber type (**D**). wo, week-old; BW, body weight; SDH, succinate dehydrogenase. I, IIA, and IIB denote type I, IIA, and IIB fibers, respectively. Data are illustrated as mean ± SEM. * and † show significantly different from LETO rats and the OLETF Sed group, respectively, *P* < 0.05, one way ANOVA. *n* = 5–6 per group.

At 12-week-old, although there were no significant differences in the muscle wet weight between the OLETF Sed and LETO rats (Fig 4A), the ratio of muscle wet weight to body weight was significantly lower in the OLETF Sed group than in LETO rats (Fig 4B). In addition, the muscle wet weight was significantly lower in the OLETF Ex group than in the OLETF Sed group. Conversely, the ratio of muscle wet weight to body weight was significantly higher in the OLETF Ex group than in the OLETF Sed group. At 20-week-old, the muscle wet weight and the ratio of muscle wet weight to body weight were significantly lower in the OLETF Sed group than in LETO rats. However, the ratio of muscle wet weight to body weight was still significantly higher in the OLETF Ex group than in the OLETF Sed group.

The gastrocnemius lateralis was composed of type I, IIA, and IIB fibers in all the groups (Fig 4C). There were no significant differences in the cross-sectional areas of type I, IIA, and IIB fibers among all the groups at 12- and 20-week-old (Fig 4D).

### Histological changes and triacylglycerol contents in the liver

**Fig 5:**
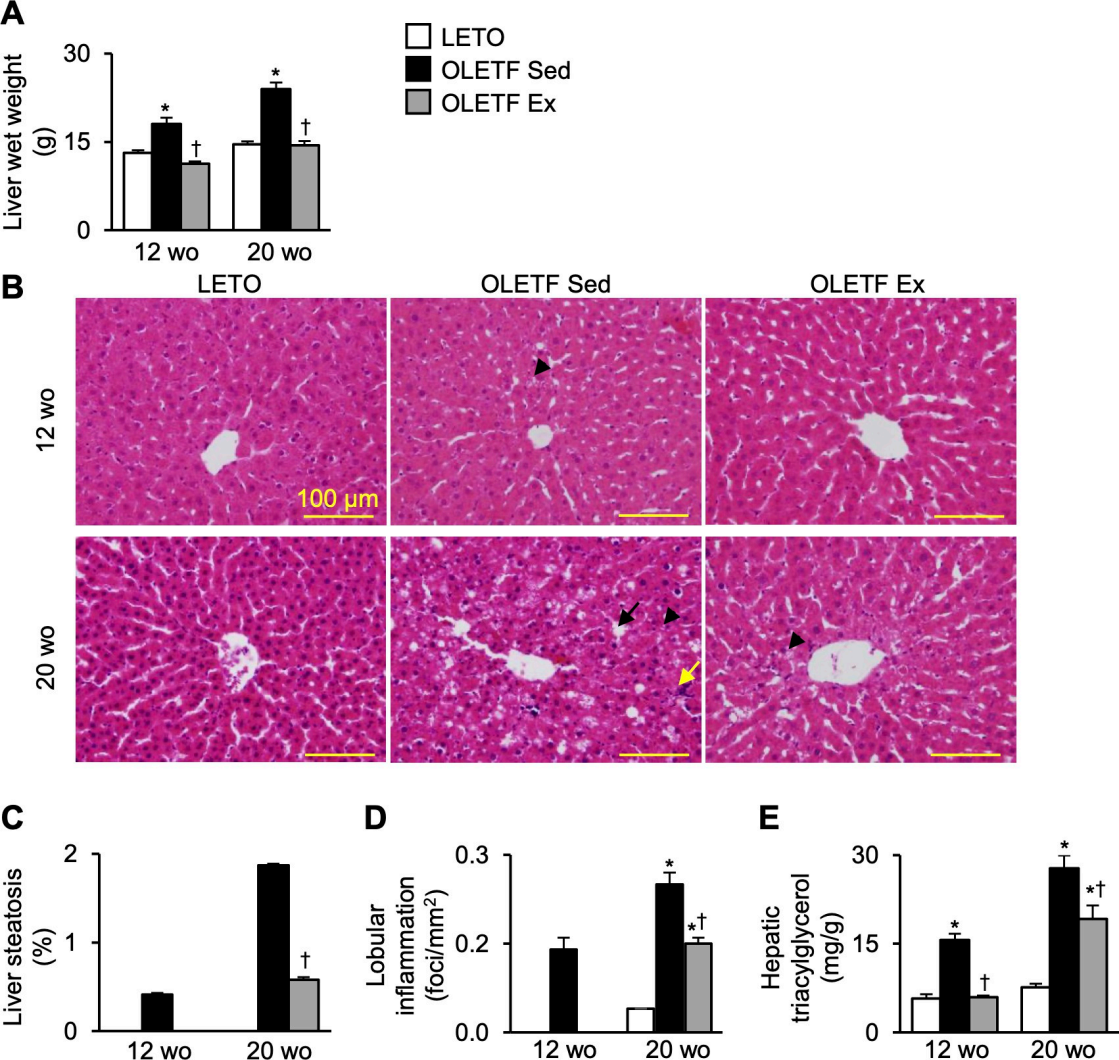
Histological changes and TAG contents in the liver. Liver wet weight (**A**), representative liver images stained with hematoxylin and eosin (**B**), percentage of liver steatosis (**C**), number of lobular inflammatory foci (**D**), and hepatic TAG contents (**E**). TAG, triacylglycerol; wo, week-old. A black arrow and a black arrowhead indicate macrovesicular steatosis and microvesicular steatosis, respectively. A yellow arrow indicates an inflammatory focus. Data are illustrated as mean ± SEM. * and † show significantly different from age-matched LETO rats and age-matched the OLETF Sed group, respectively, *P* < 0.05, one way ANOVA. *n* = 5–6 per group.

The liver wet weight was significantly higher in the OLETF Sed group than in LETO rats at 12- and 20-week-old (Fig 5A). However, at 12-week-old, the value was significantly lower in the OLETF Ex group than in the OLETF Sed group. In addition, these values were still significantly lower in the OLETF Ex group than in the OLETF Sed group at 20-week-old.

Histologically, at 12-week-old, microvesicular steatosis in the liver was observed only in the OLETF Sed group (Fig 5B). Liver steatosis (Fig 5C) and lobular inflammation (Fig 5D) were detected in the OELTF Sed group but not in the OLETF Ex group. At 20-week-old, liver histology showed microvesicular and macrovesicular steatoses scattered in the hepatic lobules in the OLETF Sed group (Fig 5B). Microvesicular steatosis with a few macrovesicular steatosis and inflammatory foci near the center of hepatic lobules were detected in the OLETF Ex group. The percentage of liver steatosis in the OLETF Ex group was significantly lower than that in the OLETF Sed group but not in LETO rats (Fig 5C). The number of inflammatory foci in the OLETF Ex group was significantly lower than that in the OLETF Sed group but significantly higher in LETO rats (Fig 5D).

Hepatic TAG content was significantly higher in the OLETF Sed group than in LETO rats at 12- and 20-week-old (Fig 5E). However, at 12-week-old, these values were significantly lower in the OLETF Ex group than in the OLETF Sed group. In addition, at 20-week-old, the hepatic TAG content in the OLETF Ex group was still significantly lower than that in the OLETF Sed group, which was significantly higher than that in LETO rats.

### Histological changes in the white adipose tissue

**Fig 6:**
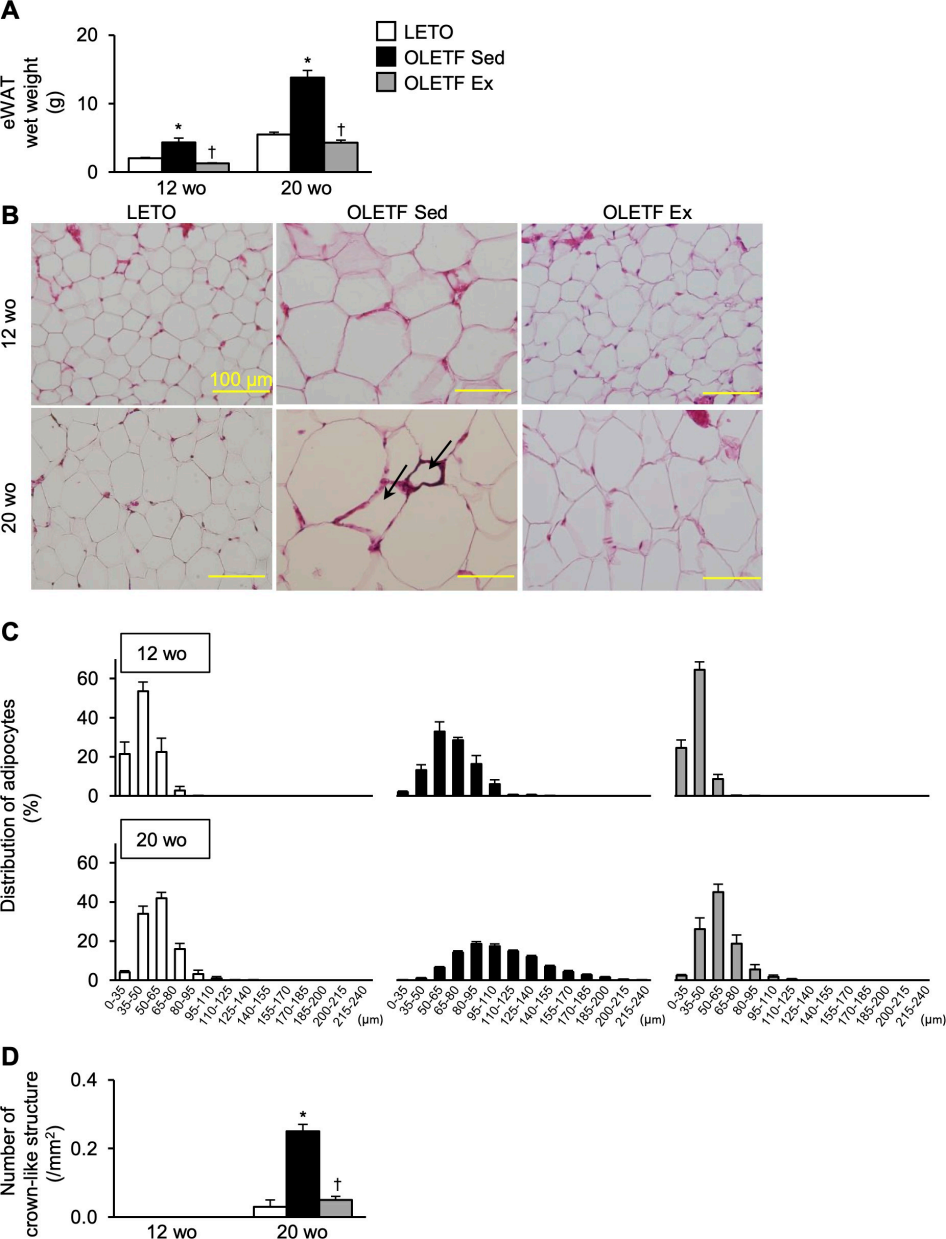
Histological changes in the epididymal white adipose tissue. eWAT wet weight (**A**), representative eWAT images stained with hematoxylin and eosin (**B**), distribution of adipocyte diameter (**C**), and number of crown-like structures (**D**). eWAT, epididymal white adipose tissue; wo, week-old. Black arrows indicate crown-liked structure. Data are illustrated as mean ± SEM. * and † show significantly different from age-matched LETO rats and age-matched the OLETF Sed group, respectively, *P* < 0.05, one way ANOVA. *n* = 5–6 per group.

The eWAT wet weight was significantly higher in the OLETF Sed group than in LETO rats at 12- and 20-week-old (Fig 6A). At 12-week-old, the value was significantly lower in the OLETF Ex group than in the OLETF Sed group. In addition, at 20-week-old, the value was still significantly lower in the OLETF Ex group than in the OLETF Sed group.

Histologically, at 12-week-old, larger adipocytes were always observed in the OLETF Sed group but rarely in the OLETF Ex group (Fig 6B). The distribution of adipocyte diameter shifted to the right in the OLETF Sed group, with diameters ranging from 95 to 155 μm in the OLETF Sed group but not in the OLETF Ex group (Fig 6C). No CLS was observed in any group (Fig 6D). From 12- to 20-week-old, an increased adipocyte size was histologically observed in all groups (Fig 6B). At 20-week-old, adipocytes with diameters ranging from 125 to 240 μm were observed in the OLETF Sed group but not in the OLETF Ex group (Fig 6C). Additionally, CLS was less abundant in the OLETF Ex group than in the OLETF Sed group, and the number of CLS was significantly lower in the OLETF Ex group than in the OLETF Sed group (Fig 6D).

### Histological changes in the brown adipose tissue

**Fig 7:**
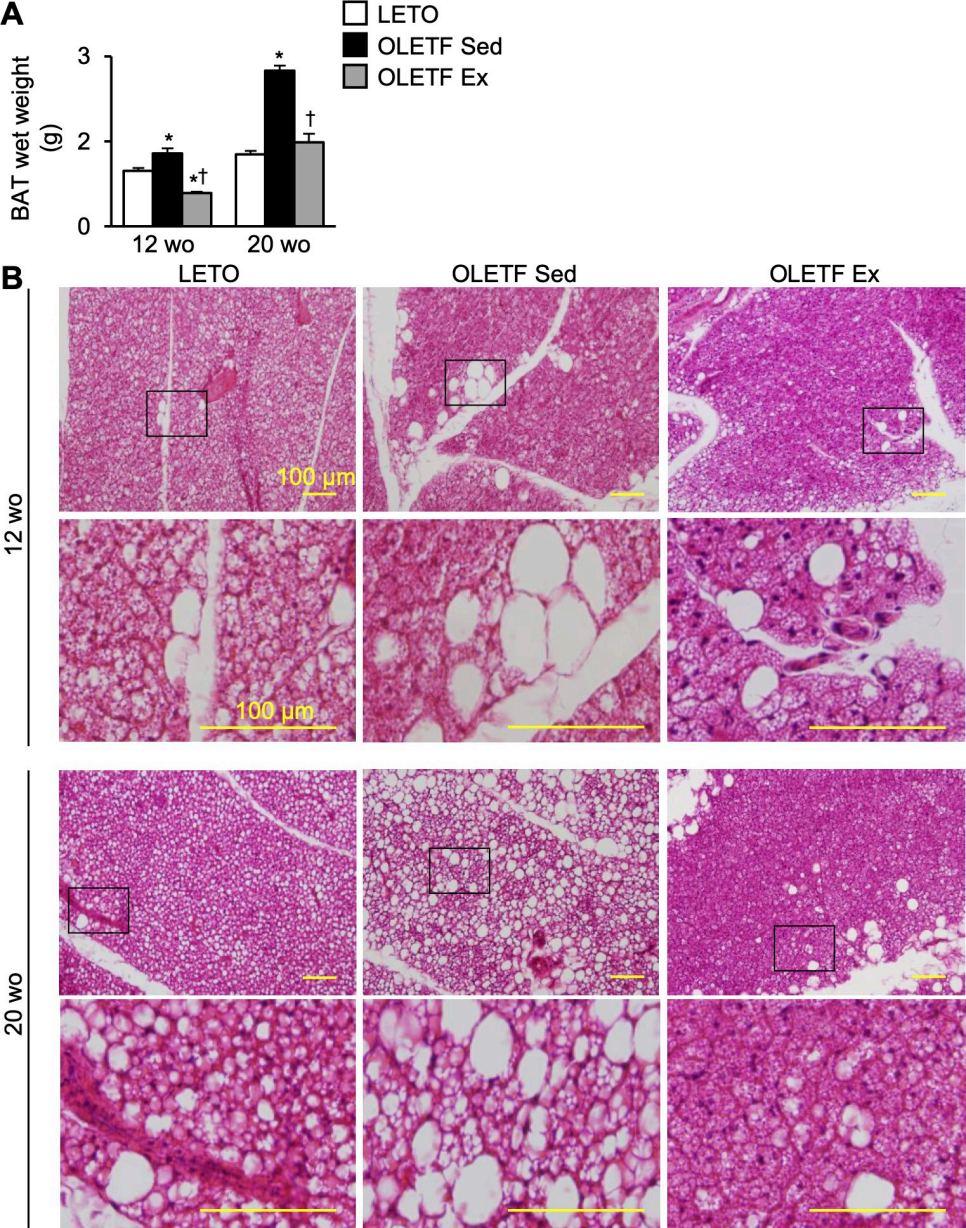
Histological changes in the brown adipose tissue. BAT wet weight (**A**) and representative BAT images stained with hematoxylin and eosin (**B**). BAT, brown adipose tissue; wo, week-old. Data are illustrated as mean ± SEM. * and † show significantly different from age-matched LETO rats and age-matched the OLETF Sed group, respectively, *P* < 0.05, one way ANOVA. *n* = 5–6 per group.

The BAT wet weight was significantly higher in the OLETF Sed group than in LETO rats at 12- and 20-week-old (Fig 7A). However, at 12-week-old, the value was significantly lower in the OLETF Ex group than in the OLETF Sed group. In addition, at 20-week-old, the value was still significantly lower in the OLETF Ex group than in the OLETF Sed group.

Histologically, BAT whitening was observed in all groups at 12-week-old, BAT whitening was observed especially in the periphery of the adipose lobule (Fig 7B). Although whitened unilocular adipocytes were more abundant and larger in the OLETF Sed group, smaller whitened and multilocular adipocytes were observed in the OLETF Ex group. At 20-week-old, the whitened unilocular adipocytes in the OLETF Sed group were evenly scattered throughout the adipose lobule, whereas the localization in the OLETF Ex group appeared to be more restricted to almost the periphery of the lobule.

## Discussion

The present study revealed that childhood exercise completely inhibited hyperphagia and subsequent obesity, including histological lipid accumulation in the liver, WAT, and BAT, during the exercise period, regardless of non-weight loss. Additionally, childhood exercise attenuated the loss of muscle mass in obesity and prevented the enlargement of pancreatic islets in diabetes. A study by Crissey *et al*. involving adult OLETF rats exercised from 12- to 20-week-old also exhibited a significant decrease in lipid accumulation in the liver, WAT, and BAT [15]. However, body weight marginally decreased compared to baseline in their study. The differences in weight loss between childhood and adulthood could be due to the developmental stages, with a steeper growth velocity in childhood. Childhood exercise inhibits the development of obesity without negatively affecting children’s growth. Although both food restriction and exercise in childhood could prevent the development of obesity, the effectiveness of food restriction could not be maintained after cessation. Shindo *et al*. found that in childhood OLETF rats both early food restriction and exercise interventions reduced body weight to the same level, however, after stopping interventions, only formerly exercised OLETF rats maintained lower both body weight and fat mass parameters compared with OLETF sedentary rats [16]. The differences between the two kinds of intervention were partly attained to the superior suppression of *de novo* lipogenesis and an enhancement of oxidizing redundant metabolic fuels of early exercise intervention [17]. These findings indicated that besides the reduction in body weight, childhood exercise could uniquely prolong its effectiveness in preventing obesity due to the inhibition of lipid accumulations in metabolic-related organs.

The present study revealed that childhood exercise maintained lower food intake during the detraining period and prevented obesity and diabetes, even though a slight glucose intolerance was found after glucose administration. In addition to maintaining higher muscle mass and smaller pancreatic islets, childhood exercise histologically inhibited lipid accumulation in the WAT and BAT after the detraining period. However, the effectiveness was only partially successful in fat accumulation and inflammation in the liver. A study by Yasari *et al*. involving adult rats with obesity, exercised for 8 weeks and then detrained for 6 weeks, showed that detraining completely reversed the effectiveness of exercise with rapid increases in intra-abdominal fat deposition, the diameter of adipocytes in the WAT, and activity of lipoprotein lipase to a level not different from that of age-matched sedentary rats [18]. Moreover, in 10-week-old mice fed a high-fat diet, although 8-week exercise reduced significantly fat mass compared, no significant difference was observed between formerly exercised mice and sedentary mice after 8-week subsequent detraining [19]. The results suggest that detraining in adulthood reverses the inhibition of lipid accumulation in the WAT induced by regular exercise, which disagrees with the results of the present study using young rats. The number of adipocytes increases during childhood and adolescence, which remains constant in adulthood [20]. However, in childhood obesity, if adipocytes in the WAT are enlarged with excessive food consumption and hypertrophy exceeds the critical limitation, overwhelming hypertrophy inhibits an increase in adipocyte number and simultaneously secretes pro-inflammatory cytokines in the presence of CLS, contributing to systemic inflammation and peripheral insulin resistance [21, 22]. In the present study, childhood exercise inhibited hyperphagia during the exercise period, resulting in a normal distribution of adipocyte diameters immediately after the exercise period. Therefore, OLETF rats in the Ex group could show a normal increase in adipocyte number during the exercise period from 4- to 12-week-old. The number of adipocytes could compensate for hypertrophy during the detraining period and inhibit inflammation, which contributes to maintaining normal insulin sensitivity.

Whole metabolic functions during the detraining period may also contribute to the inhibition of obesity and diabetes from childhood to adolescence. Physiologically, studies found that daily energy expenditure increase in the vicinity of puberty and remains stable during adulthood [23, 24]. Thermogenesis in the BAT contributes to an increase in resting metabolic rate early in life [25–27]. However, its activity declines with aging, which is histologically known as BAT whitening. In addition to aging, obesity accelerates BAT whitening. A study in a model of rats with obesity in the prediabetes stage showed lipid accumulation in the BAT with a positive energy balance [28]. The whitening process decreases mitochondrial functions with the downregulation of uncoupling protein-1 and vascularity in the BAT, thereby impairing thermogenesis and resting metabolic rate [29]. The present study showed that childhood exercise inhibited BAT whitening after the detraining period, suggesting increased lipid metabolism during childhood and adolescence. The resting metabolic rate, especially in adults, depends on heat production in the BAT and skeletal muscle mass, almost equivalent to lean body mass. Compared with their lean counterparts, in humans and rats with obesity, children with obesity show normal or increased lean body mass, but adults with obesity show decreased lean body mass [16, 30, 31]. In the present study, the ratio of muscle wet weight to body weight was higher in the OLETF Ex group than in the OLETF Sed group at 20-week-old, which suggested that childhood exercise maintained higher metabolic-related organs after the detraining period. Therefore, lipid metabolic function after the detraining period could be increased by inhibiting BAT whitening and maintaining skeletal muscle mass during childhood exercise. Whitening process leads to a reduction in uncoupling protein expression and activity of BAT [29], which negatively affects lipolysis and FFA oxidation [32]. Additionally, skeletal muscle was found positively correlated with the two important enzymes in circulating lipid metabolism (*i*.*e*., lipoprotein lipase and glycosylphosphatidylinositol anchored high-density lipoprotein binding protein 1) [33]. In obesity, a low resting metabolic rate contributes to a positive energy balance [34]. Therefore, lower food intake could inhibit BAT whitening and increase energy consumption while maintaining skeletal muscle mass.

After the detraining period, childhood exercise diminished food intake and lipid accumulation in adipose tissue, even though it was only partially suppressed by inflammation and lipid accumulation in the liver. It is known that childhood exercise attenuates lipid accumulation in the liver, whereas the effectiveness of adult exercise on lipid accumulation in the liver waned after the detraining period. Specifically, in a study by Yasari *et al*. involving adult rats with obesity, hepatic TAG content in formerly exercised rats increased to a level significantly higher than that in sedentary rats after 6 weeks of detraining [18]. Lipid accumulation in the liver stems from two sources: 75% from non-hepatic FFAs (*i*.*e*., from adipose tissues and diet) and 25% from *de novo* lipogenesis [35]. The partial success of childhood exercise in inhibiting lipid accumulation in the liver after detraining is likely due to *de novo* lipogenesis. However, the present study showed that childhood exercise completely inhibited lipid accumulation in adipose tissues, suggesting a reduction in circulating FFAs in the liver. In contrast to childhood exercise, the effect of adulthood exercise is often incomplete on lipid accumulation in adipose tissues [36, 37]. Incomplete effectiveness of adulthood exercise on lipid accumulation in adipose tissues could contribute to lipid reaccumulation in the liver after the detraining period. In contrast, childhood exercise completely inhibits lipid accumulation in adipose tissues, resulting in partial preservation of lipid accumulation in the liver after the detraining period.

The present study revealed that long-term childhood exercise attenuated body weight gain, glucose intolerance, and lipid accumulation in the organs carried over to adulthood. Nevertheless, this study had some limitations. First, the study focused on the imaging and variability of some indirect reflections of metabolism. Future studies on the molecular mechanisms of lipid metabolism in each organ after childhood detraining are needed to clarify this. Second, the study participants were completely obese because of genetic defects. Additionally, a study on 5-week-old non-obese Wistar rats found that 10-week detraining following 10-week exercise led to no significant changes in body weight compared with non-exercise rats [38]. Therefore, the interpretation of the results for other models requires specific studies for each specific obese model, and future studies are needed to clarify whether there are differences in detraining in both obese and non-obese childhood models. Third, the follow-up time was only 8 weeks post-exercise in OLETF rats; thus, it was impossible to confirm an effect on weight and metabolism beyond this time. However, obesity and metabolic disorders in individuals with genetic obesity require regular dietary and exercise interventions throughout childhood to prevent them in adulthood. In addition to childhood obesity, gestational diabetes mellitus could pose risk in the development of metabolic disorders and obesity in offspring. The present study showed the merits of childhood exercise in preventing obesity and its related consequences. It is interesting to repeat the experiment in both maternal individuals and their offspring to clarify the advantages of exercise in full prevention of obesity. In conclusion, the results suggested that long-term childhood exercise was effective in the long-term prevention of obesity and diabetes, possibly due to the inhibition of both BAT whitening and reduction of skeletal muscle mass, and adequate inhibition of hyperphagia. Additionally, childhood exercise inhibited lipid accumulation in the WAT and BAT and attenuated lipid accumulation in the liver after the detraining period, suggesting the importance of exercise during childhood and adolescence in inhibiting hyperphagia-induced lipid accumulation in metabolic-related organs in adulthood.

## Supporting information

S1 Fig Wheel running parameters. Total running duration per day (**A**), total running distance per day (**B**), average running velocity in a day (**C**), and maximum running velocity in a day (**D**). Data are illustrated as mean ± SEM. *n* = 12.
(TIF)

S1 Dataset (XLSX)
