# Supplementary figures and images for: Effects of long-term childhood exercise and detraining on lipid accumulation in metabolic-related organs
Source: PLoS One. 2022 Jun 24;17(6):e0270330. doi: 10.1371/journal.pone.0270330 (PMC9231767; doi:10.1371/journal.pone.0270330)

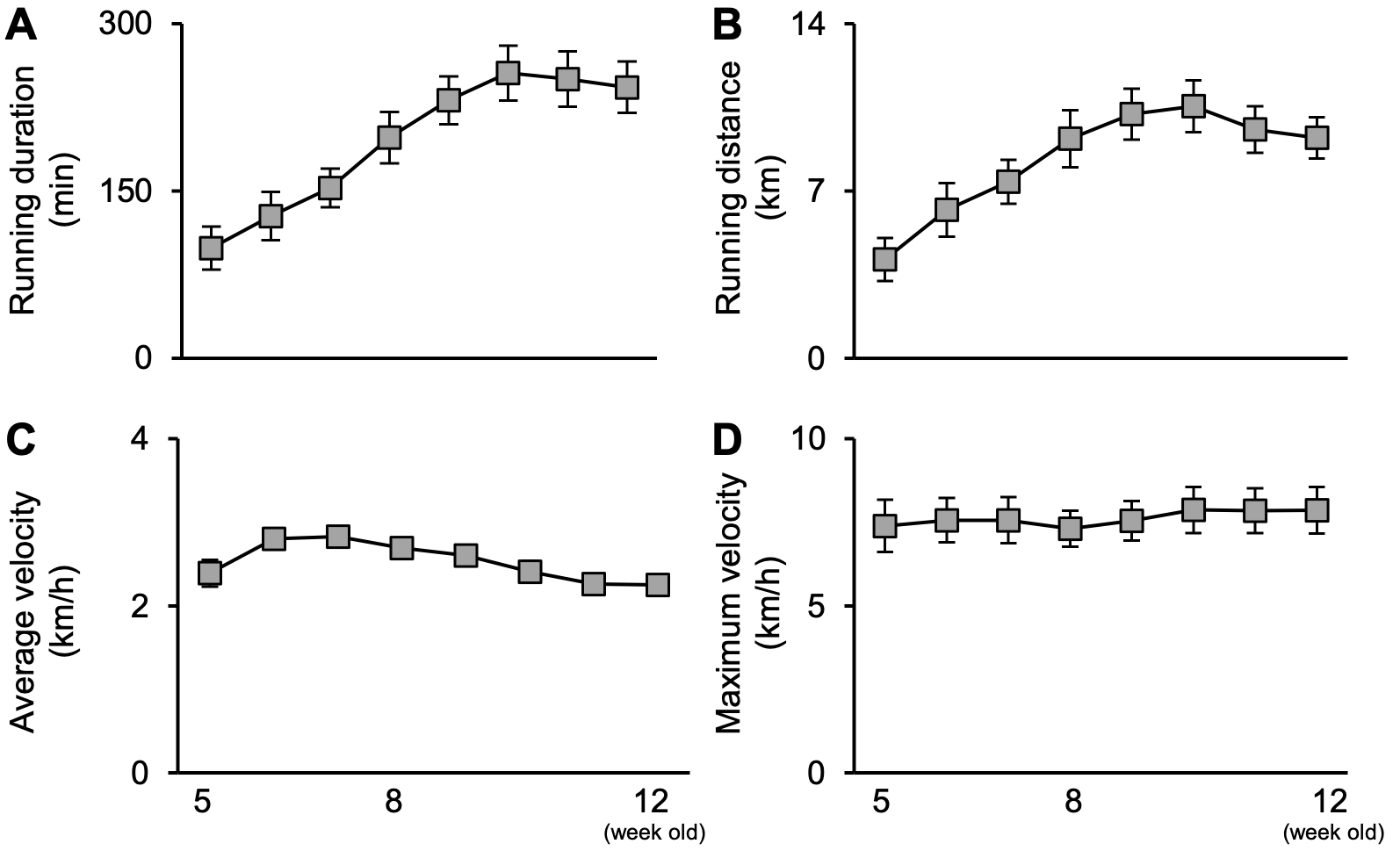

Supplement: S1 Fig — Total running duration per day (A), total running distance per day (B), average running velocity in a day (C), and maximum running velocity in a day (D). Data are illustrated as mean ± SEM. n = 12. (TIF) [file pone.0270330.s001.tif]
